# Supplementary material for: fMRI-based prediction of eye gaze during naturalistic movie viewing reveals eye-movement-related brain activity
Source: Psychoradiology. 2026 Jun 26;6:kkag026. doi: 10.1093/psyrad/kkag026 (PMC13421079; doi:10.1093/psyrad/kkag026)

**Supplementary Materials for “fMRI-Based Prediction of Eye Gaze During Naturalistic Movie Viewing Reveals Eye-Movement–Related Brain Activity”**

Le Gao^1^, Zhi Wei^1^, Bharat B. Biswal^2^, Xin Di ^2*^

1. Department of Computer Science, New Jersey Institute of Technology, Newark, NJ, 07102, USA

2. Department of Biomedical Engineering, New Jersey Institute of Technology, Newark, NJ, 07102, USA

The supplementary materials provide additional methodological validation and quality control (QC) analyses supporting the main manuscript. First, to confirm the integrity of our preprocessing pipeline, we present representative QC reports demonstrating successful eye-voxel extraction and alignment across all three datasets (Figure S1). Second, to address the potential influence of ground-truth measurement noise on individual-level decoding accuracy, we quantify the relationship between camera-based eye-tracking data quality (data retention and spatial precision) and model prediction accuracy in the Natural Viewing dataset (Figure S2). Third, we assess the utility of the model's unsupervised Predicted Error (PE) metric by examining its relationship to both spatial accuracy (Mean Absolute Error) and correlational performance (Figure S3), further characterizing the boundaries of individual-level gaze decoding. Fourth, we provide a visual demonstration of the eye-tracking preprocessing pipeline, illustrating the raw recording artifacts and the subsequent effects of linear interpolation on missing gaze segments (Figure S4). Finally, we evaluate the relationship between individual-to-group gaze consistency and camera-to-model decoding performance to untangle the relative contributions of ground-truth tracking noise versus intrinsic model limitations under a zero-shot setting (Figure S5).

**Supplementary Figure S1. Quality control of eye-voxel extraction across datasets.** Representative quality control (QC) visual reports generated by the DeepMReye preprocessing pipeline. Panels display the successful alignment and extraction of eye voxels for a representative participant from the (a) Natural Viewing (NV), (b) Healthy Brain Network (HBN), and (c) Partly Cloudy (PC) datasets.

**
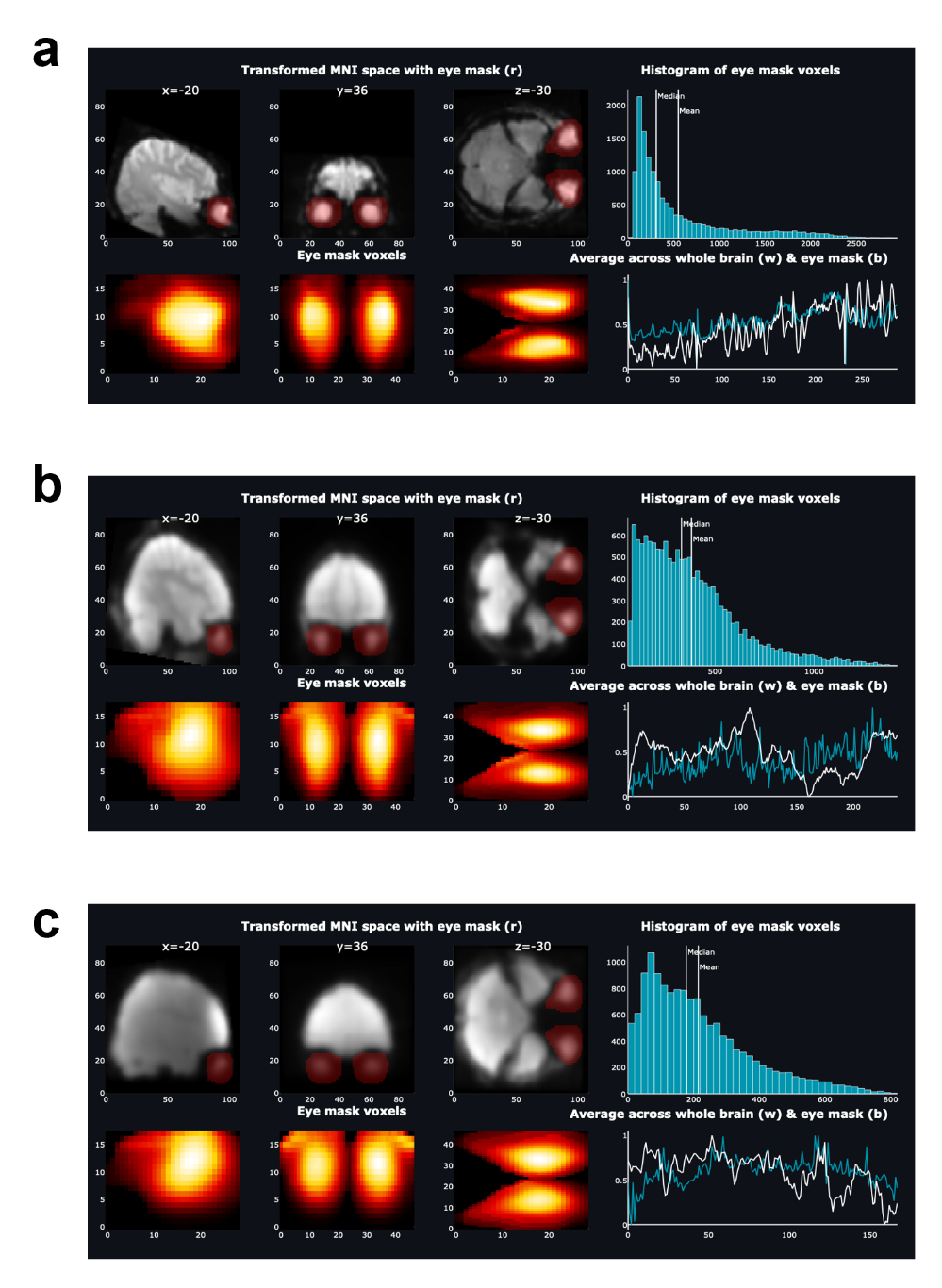
**

**Figure S2. Relationship between ground-truth eye-tracking data quality and model prediction accuracy in the Natural Viewing (NV) dataset.**

(a-b) Data from the *Despicable Me* (DM) stimulus. Panel (a) shows the association between the proportion of valid eye-tracking samples retained per participant and their DeepMReye prediction accuracy (Pearson’s *r*). Panel (b) shows the association between eye-tracking spatial precision—quantified as the root mean square (RMS) of sample-to-sample gaze displacement—and prediction accuracy.

(c-d) Parallel analyses for *The Present* (TP) stimulus. Panel (c) displays prediction accuracy as a function of the valid data proportion, and panel (d) shows accuracy as a function of RMS error. Shaded regions represent 95% confidence intervals.

**
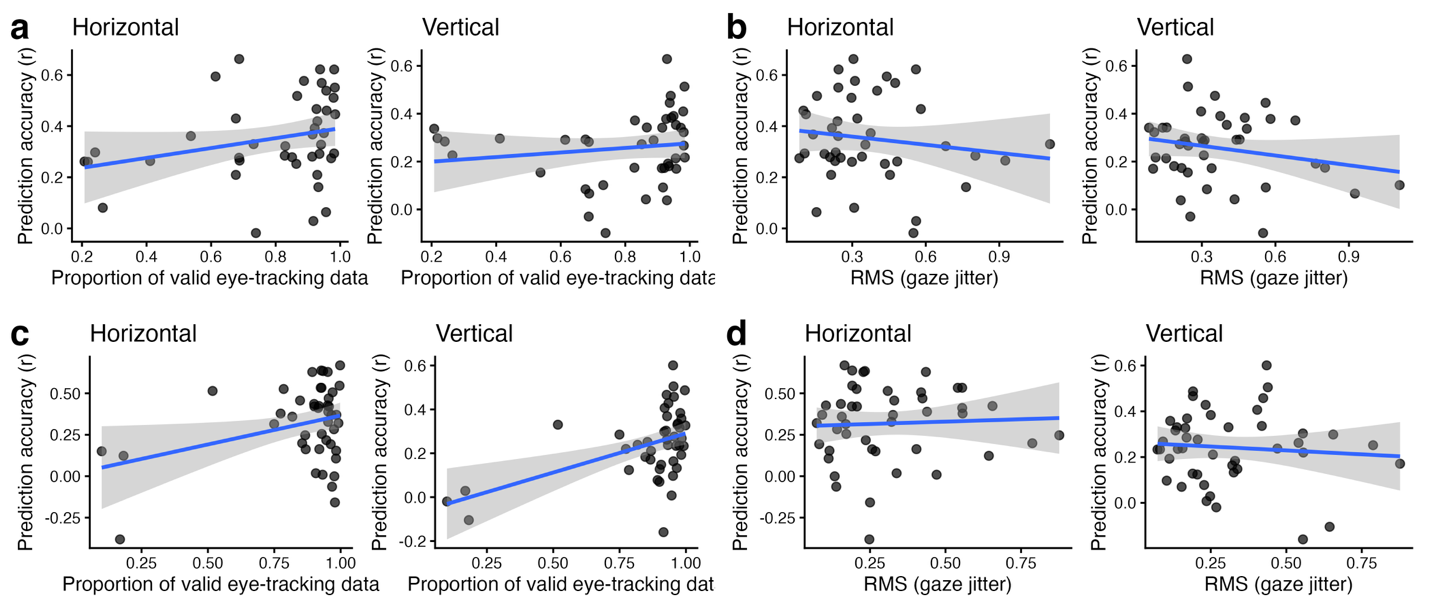
**

**Supplementary Figure S3. Relationship between the model's Predicted Error (PE) and gaze prediction performance in the Natural Viewing dataset.**

Model performance was evaluated using Pearson correlation (*r*) and Mean Absolute Error (MAE) across participants, stratified into low-PE and high-PE groups using a median split.

(a) Pearson correlation in the *Despicable Me* (DM) dataset for horizontal and vertical gaze components. Two sample t-tests revealed a significant difference for the vertical component, with higher values in the high-PE group.

(b) MAE in the DM dataset, showing no significant differences between PE groups.

(c) Pearson correlation in *The Present* (TP) dataset, showing no significant differences between groups for either gaze component.

(d) MAE in the TP dataset, demonstrating significantly higher spatial error in the high-PE group for both horizontal and vertical components.

**
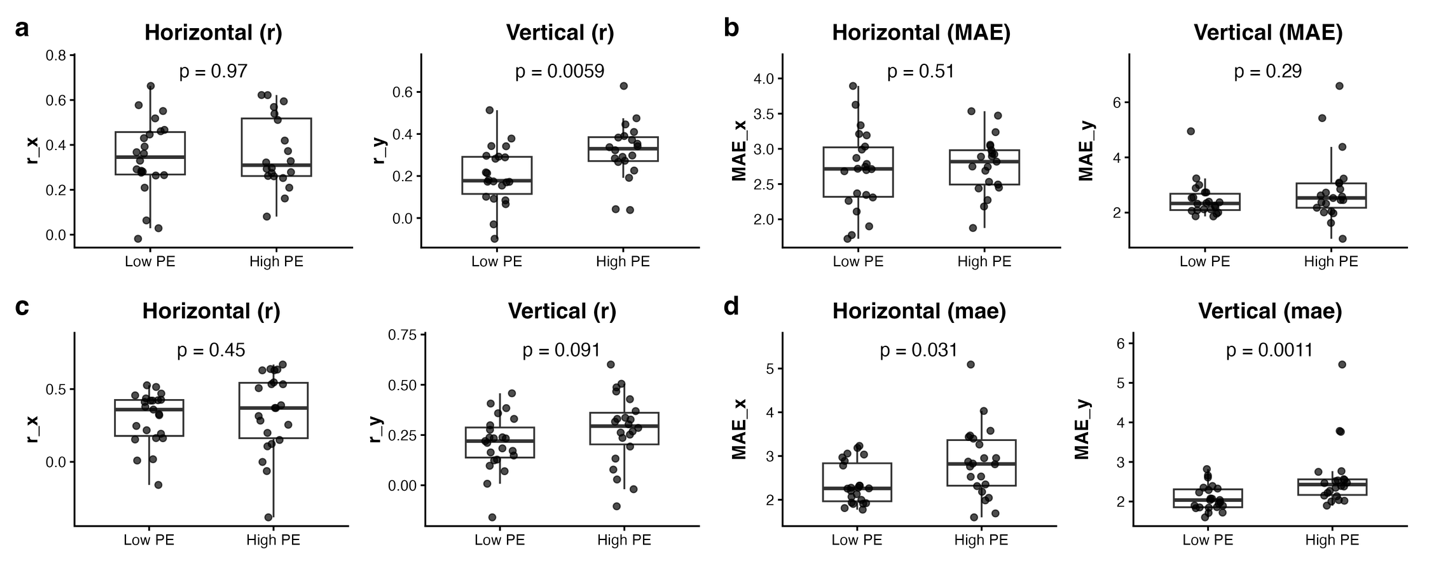
**

**Supplementary Figure S4. Representative example of preprocessing and interpolation effects on gaze trajectories.** Time-series plots show individual-level horizontal and vertical gaze coordinates before and after data cleaning. The raw camera recordings demonstrate prominent missing values caused by blinks or tracking dropouts, while the processed data illustrate the effect of our pipeline, where these missing segments have been resolved via linear interpolation and temporally aligned to the fMRI TR resolution.

**
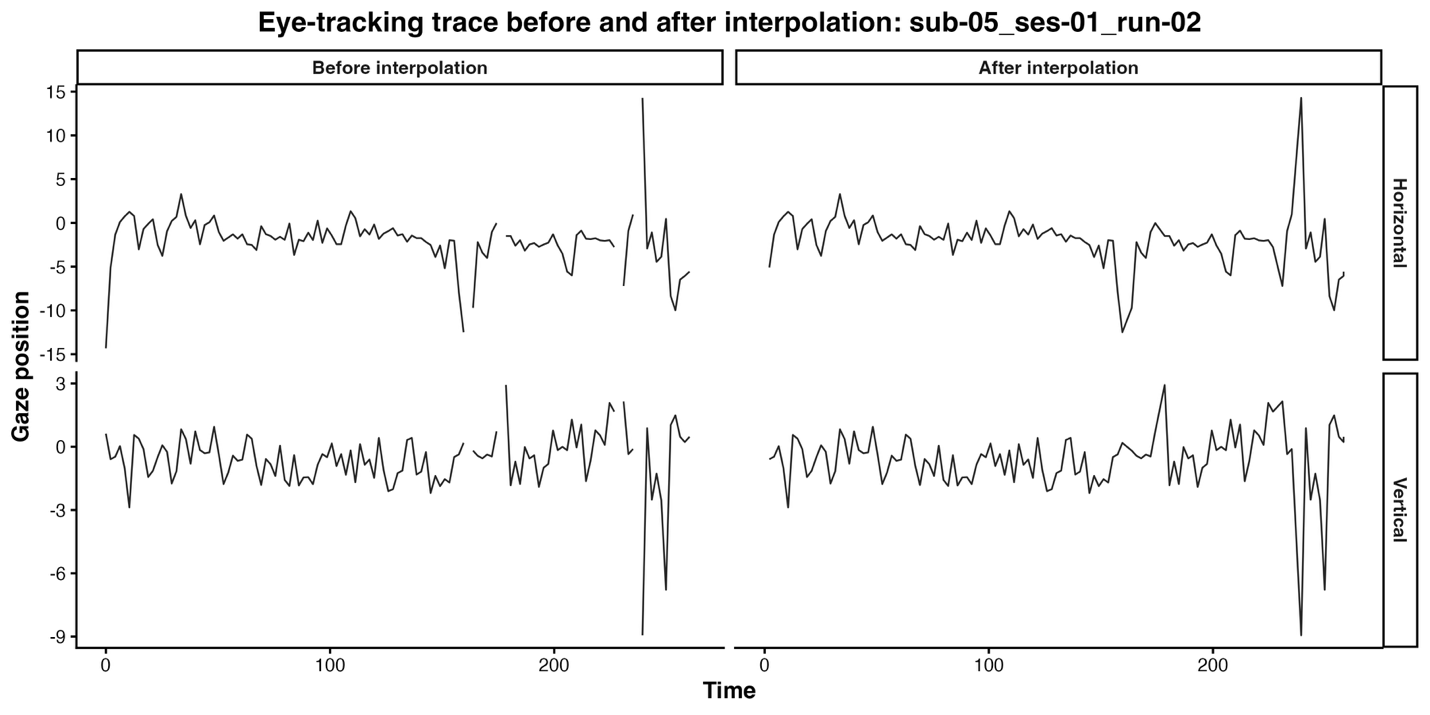
**

**Supplementary Figure S5. Relationship between camera-to-model performance and subject-to-group gaze consistency.** Scatter plots illustrate the association between an individual's gaze consistency (Pearson correlation with the leave-one-out group-averaged eye-tracking trajectory) and the model's decoding performance (Pearson correlation and Mean Absolute Error [MAE]) in the Natural Viewing dataset. In the horizontal dimension (left), higher subject-to-group consistency significantly correlates with higher camera-to-model correlation (*r* = 0.47, *p* = 0.0015) and lower MAE (*r* = −0.40, *p* = 0.0076). In the vertical dimension (right), these relationships follow a similar direction but are weak and non-significant (*p* > 0.05). These results demonstrate that while individual behavioral variations or tracking noise partially influence horizontal decoding accuracy, they do not fully account for the lower individual-level performance observed in the zero-shot setting.


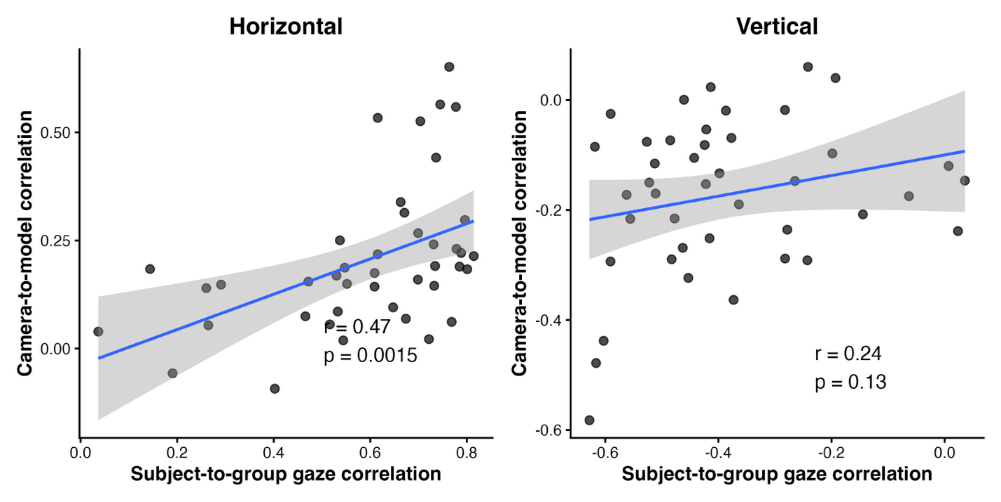


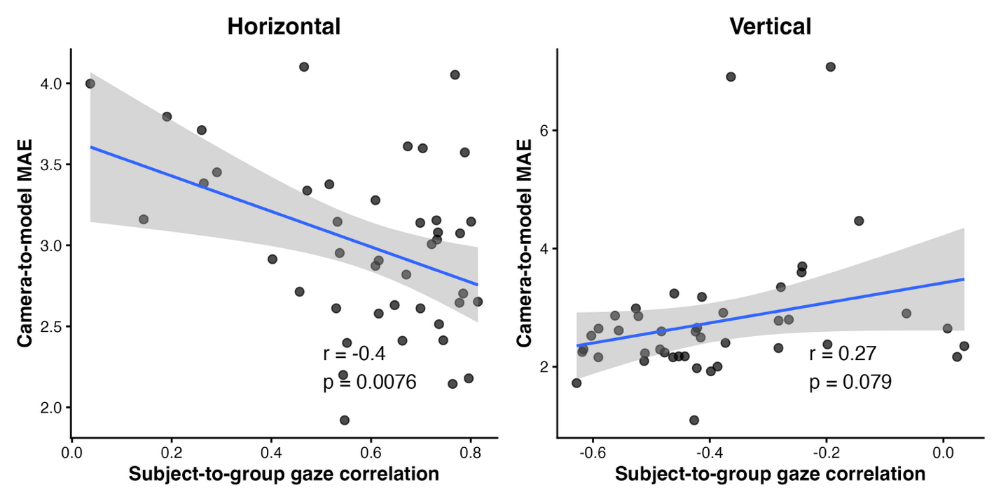

Supplement: kkag026_Supplemental_File [file kkag026_supplemental_file.docx]
